# Timely diagnosis and management of *Quambalaria cyanescens*-induced peritoneal dialysis peritonitis: A rare case highlighting the role of galactomannan testing

**DOI:** 10.1016/j.mmcr.2025.100697

**Published:** 2025-02-11

**Authors:** Baramett Somtha, Kanjana Tianprasertkij, Supattra Promngam, Thunvarat Saejew, Talerngsak Kanjanabuch

**Affiliations:** a Faculty of Medicine, Chulalongkorn University, Bangkok, Thailand; b Dialysis Unit, Sakaeo Crown Prince Hospital, Sakaeo, Thailand; c Center of Excellence in Kidney Metabolic Disorders, Faculty of Medicine, Chulalongkorn University, Bangkok, Thailand; d Division of Nephrology, Department of Medicine, Faculty of Medicine, Chulalongkorn University, Bangkok, Thailand; e CAPD Excellence Center, King Chulalongkorn Memorial Hospital, Bangkok, Thailand

**Keywords:** Fungal peritonitis, *Quambalaria cyanescens*, Peritoneal dialysis, Peritonitis, *Quambalaria*, Galactomannan

## Abstract

Fungal peritonitis in peritoneal dialysis (PD) presents significant challenges. We report the second *Quambalaria cyanescens*-related PD peritonitis in a 53-year-old male. Negative bacterial cultures and a positive galactomannan (GM) index in both PD effluent (PDE) (0.65) and serum (0.98) prompted early PD catheter removal on day 5. Molecular sequencing confirmed *Q. cyanescens*, with antifungal susceptibility testing revealing resistance to azoles and echinocandins but susceptibility to amphotericin B and isavuconazonium. Treatment with amphotericin B and voriconazole resolved symptoms, with no relapses during a two-year follow-up. This case highlights GM testing's critical role in guiding catheter removal and adherence to the 2022 ISPD Peritonitis Guidelines, ensuring favorable outcomes for rare fungal PD infections.

## 1 Introduction

Peritonitis is a serious complication in peritoneal dialysis (PD) patients, accounting for modality switches to hemodialysis (HD) in 29–60 % of cases [1], significantly affecting patient outcomes and increasing healthcare costs. While bacterial infections predominate, fungal peritonitis constitutes about 1–3 % of cases [2] and is associated with significantly higher morbidity and mortality rates [3]. Among fungal pathogens, infections caused by *Quambalaria cyanescens*, a rare basidiomycete fungus, are exceedingly uncommon. To date, only one documented case of PD-associated peritonitis caused by *Q. cyanescens* has been reported in the literature. While the case showed recovery following antifungal therapy and a short-term follow-up of two months after treatment, the delayed catheter removal by two months raises uncertainties about its long-term outcomes [4].

In this report, we present the first documented case of *Q. cyanescens*-related PD peritonitis in Thailand. Uniquely, this case demonstrates the pivotal role of galactomannan (GM) testing in guiding timely PD catheter removal, an approach not previously reported for this organism. With a two-year follow-up, our findings confirm favorable long-term outcomes when strict adherence to the 2022 ISPD Peritonitis Guidelines is maintained [5]. This case highlights the importance of early diagnosis and intervention in managing rare fungal infections in PD patients.

## 2 Case presentation

**Fig. 1:**
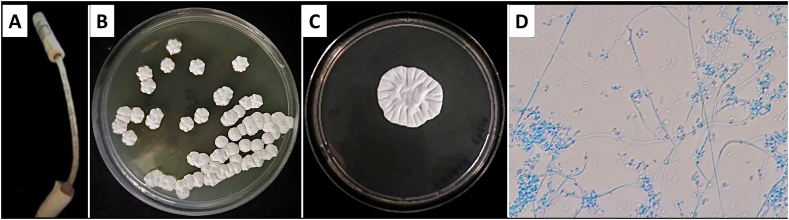
The intraluminal colonization of the PD catheter specimen [A] was cultured in Sabouraud Dextrose Agar (SDA). *Quambalaria cyanescens* exhibited growth in the form of white colonies on day 4 [B] and in a restricted, cerebriform appearance on day 7 [C]. Microscopic examination under lactophenol cotton blue stain at ×400 magnification revealed pseudohyphal budding patterns characteristic of the pathogen, including primary conidia and secondary conidia.

A 53-year-old Thai male farmer with a history of type 2 diabetes, hypertension, dyslipidemia, and kidney failure had been undergoing continuous ambulatory peritoneal dialysis (CAPD, 2L x 4 exchanges/day) for two years. His PD history included a prior episode of culture-negative peritonitis in July 2022, successfully treated with empiric antibiotics. Two months later, he presented to Sakaeo Crown Prince Hospital with a one-day history of intermittent throbbing abdominal pain, fever, chills, and eight episodes of diarrhea (Day 0). Examination revealed abdominal tenderness and brown-black intraluminal colonization within the PD catheter (Fig. 1A).

Initial analysis of the PD effluent (PDE) revealed a leukocyte count of 374 cells/μL, with 61 % neutrophils, indicating possible bacterial peritonitis. Given the patient's systemic presentation and residence in a melioidosis-endemic area [6], empiric therapy was initiated with intraperitoneal cefazolin (1 g) alongside intravenous ceftazidime (2 g every 8 hours) and metronidazole (500 mg every 8 hours). Despite this aggressive approach, the patient's clinical symptoms persisted, and bacterial cultures of the PDE remained negative, necessitating further investigation.

A positive GM index (0.65; cutoff ≥0.5) in the PDE raised suspicion of a fungal etiology. Serum GM was also positive (0.98), further supporting the diagnosis. GM testing was performed using the Platelia Aspergillus EIA test kit (Bio-Rad Laboratories, France). Based on these findings and visualization of intraluminal colonization within the PD catheter, catheter removal was performed on day 5. Fungal cultures from PDE and PD catheter specimens initially grew whitish, circular, and smooth colonies on Sabouraud Dextrose Agar (SDA). By day 7, they developed a cerebriform (brain-like) appearance with a furrowed surface (Fig. 1B and C). Microscopic examination with lactophenol cotton blue stain revealed pseudohyphal budding patterns, with both primary and secondary conidia (Fig. 1D). Molecular sequencing using universal primers (NS1/NS4 for SSU and NL1/NL4 for LSU) confirmed the pathogen as *Q. cyanescens* with 100 % identity and 100 % query coverage (accession numbers: KT186108.1 and MN162209.1, respectively).

The patient was switched to antifungal therapy, initially receiving intravenous amphotericin B (0.8 mg/kg/day) for three days, followed by oral voriconazole (6 mg/kg twice daily) for 14 days. Antifungal susceptibility testing was performed using the Broth Dilution Antifungal Susceptibility Testing of Yeasts Method (CLSI, Wayne, PA) [7]. The isolate was susceptible to amphotericin B (1 μg/mL) and isavuconazonium (1 μg/mL), while resistant to voriconazole (1 μg/mL), posaconazole (1 μg/mL), fluconazole (>64 μg/mL), itraconazole (4 μg/mL), and caspofungin (>16 μg/mL).

Post-treatment, serum GM levels normalized (0.54 at 14 days), and remained negative on follow-up (0.30 at 1 month, 0.35 at 2 months). Due to the severity of the infection, the patient was transitioned permanently to maintenance hemodialysis. No relapses of peritonitis were observed during the two-year follow-up period.

Etiological investigation revealed that despite a farming background, the patient denied recent agricultural activities or plant exposure. Instead, he admitted to improper hygiene practices during PD exchanges, suggesting contamination through external sources. Comprehensive education on sterile techniques was provided to the patient to minimize future risks.

## 3 Discussion

This case represents the second documented instance of *Q. cyanescens*-related PD peritonitis and highlights the diagnostic and therapeutic challenges posed by fungal infections in PD patients. The use of GM testing in both PDE and serum proved instrumental in guiding timely catheter removal and treatment initiation.

*Q. cyanescens*, a member of the Quambalariaceae family, is a rare basidiomycete fungus comprising five species: *Q. cyanescens, Q. pitereka, Q. eucalypti, Q. coyrecup,* and *Q. simpsonii* [[8], [9], [10]]. While primarily known as plant pathogens, these fungi have emerged as opportunistic human pathogens, particularly in immunocompromised individuals [11,12]. A previous report of *Q. cyanescens*-related PD peritonitis described a favorable outcome despite a two-month delay in catheter removal; however, the short follow-up period left uncertainties regarding the long-term prognosis [4]. In contrast, our case highlights the importance of timely catheter removal, guided by positive GM results and direct visualization of fungal colonization, to ensure sustained long-term clinical recovery.

Our findings emphasize GM testing as a valuable adjunct to conventional diagnostics. This is the first report documenting positive GM results in both PDE and serum in *Q. cyanescens*-related peritonitis, reinforcing its utility in guiding clinical decision-making [[13], [14], [15]]. Notably, the patient's GM index declined after antifungal therapy, with PDE GM at 0.65 and serum GM at 0.98 before catheter removal, decreasing to 0.54 at 14 days, 0.30 at 1 month, and 0.35 at 2 months, correlating with infection resolution. Timely catheter removal, performed within five days of the onset of peritonitis in this case, aligns with the 2022 ISPD Peritonitis Guidelines [5] and likely contributed to the favorable outcome. Delayed catheter removal in fungal peritonitis significantly increases mortality, with rates rising from 19 % (removal within 24 hours) to 94 % (removal beyond one month) [3,16]. This reinforces the necessity of early intervention.

Molecular sequencing was instrumental in confirming the identity of *Q. cyanescens*, demonstrating the importance of advanced diagnostics in identifying rare fungal pathogens that may not be readily identifiable on standard culture media [17,18]. Antifungal susceptibility testing revealed susceptibility to amphotericin B and isavuconazonium but resistance to azoles and echinocandins, necessitating a carefully selected antifungal regimen [19,20]. Intravenous amphotericin B followed by oral voriconazole achieved complete symptom resolution, yet the severity of the infection warranted permanent transition to hemodialysis. No relapse was observed during the two-year follow-up period.

Importantly, hygiene lapses during PD exchanges were identified as the most likely source of contamination. While the patient had no recent agricultural exposure, improper exchange techniques posed a significant risk for external contamination. This underscores the critical role of patient education in maintaining sterile PD practices and preventing recurrence [21,22].

In conclusion, this case illustrates the emerging clinical significance of *Q. cyanescens* in PD-associated peritonitis and reinforces GM testing as a valuable decision-making tool for timely catheter removal. Adherence to the 2022 ISPD Peritonitis Guidelines, integration of molecular diagnostics, and early targeted therapy were key factors in achieving a favorable long-term outcome. This case further emphasizes the need for increased awareness, improved diagnostic approaches, and enhanced patient education to mitigate the risks of fungal peritonitis in PD patients.

## CRediT authorship contribution statement

**Baramett Somtha:** Writing – review & editing, Writing – original draft, Conceptualization. **Kanjana Tianprasertkij:** Data curation. **Supattra Promngam:** Data curation. **Thunvarat Saejew:** Writing – review & editing. **Talerngsak Kanjanabuch:** Writing – review & editing, Funding acquisition, Formal analysis, Conceptualization.
